# Preparation and evaluation of the exotoxin A nano-gold conjugate as a vaccine candidate for *Pseudomonas aeruginosa* infections

**DOI:** 10.22038/IJBMS.2021.58367.12960

**Published:** 2021-10

**Authors:** Masoumeh Abbasi, Asghar Tanomand, Farshid Kafilzadeh, Samaneh Zolghadri, Hasan Hosainzadegan

**Affiliations:** 1 Department of Microbiology, Jahrom Branch, Islamic Azad University, Jahrom, Iran; 2 Department of Microbiology, Maragheh University of Medical Sciences, Maragheh, Iran

**Keywords:** Exotoxin A, Gold nanoparticles, Immunization, Pseudomonas aeruginosa, Vaccine candidate

## Abstract

**Objective(s):**

*Pseudomonas aeruginosa* is an opportunistic pathogen that is an important cause of nosocomial infections. This bacterium produces various virulence factors, among which exotoxin A is significantly involved in mortality and morbidity. In this study, we evaluated the immunogenicity of native exotoxin A extracted from the *P. aeruginosa *and its conjugation with gold nanoparticles in the animal model.

**Materials and Methods:**

Exotoxin A was first extracted and purified from the culture medium of *P. aeruginosa* PAO1 by selective precipitation and dialysis. The gold nanoparticles were prepared using the Turkevich method and conjugated to the prepared exotoxin A by electrostatic force. The size and conjugation were confirmed using electron microscopy and Fourier transform infrared spectrometry (FTIR), respectively. The immunogenicity of prepared ExoA-gold nanoparticles was investigated in the mice model.

**Results:**

The results indicated that nano-gold particles can be conjugated to the native exotoxin A with high efficiency. Immunogenicity investigation demonstrated that antibody titers produced against native exotoxin A and its conjugate to nano-gold particles are significant in a mouse model (*P*<0.005). Moreover, significant protection against 2×LD50 *P. aeruginosa* infection was observed in animals immunized with nano-gold-exotoxin A as compared with control groups (*P*=0.00).

**Conclusion:**

Our study indicated that exotoxin A can be produced with acceptable purity in the laboratory, and conjugated to gold nanoparticles. Based on these results nano-gold-exotoxin A conjugate is highly immunogenic and can be considered a potential vaccine candidate for *P. aeruginosa* infections.

## Introduction

*Pseudomonas aeruginosa *is a gram-negative, obligate aerobic bacterium, which can be isolated from plants, soil, water, and warm humid environments (1). This bacterium is an opportunistic pathogen that infects humans and animals and is a common cause of nosocomial infections in patients with burns, cancer, cystic fibrosis, and immune-deficient individuals (2). 

Various virulence factors including surface factors (e.g., pilus, flagella, lipopolysaccharide-derived polysaccharide layers), type III secretory system, and other secreted proteins are involved in the pathogenesis of *P. aeruginosa* (3). The virulence factors of *P. aeruginosa* are divided into two groups: extracellular and intracellular (4). Exotoxin A is an extracellular virulence factor, with high antigenic and cytotoxicity properties, which inhibit protein synthesis through ADP-ribosylation (5, 6). The antigenic properties, stimulating a humoral immune response, stability of induced antibodies, as well as features such as inhibition of protein synthesis and down-regulation of MHC-1 molecules on liver cells have made exotoxin A an ideal candidate for vaccine production (5, 2).

In recent years, nanoparticles of various elements such as gold, silver, and zinc have been used for treatment of bacterial, fungal, and viral infections (7, 8). These nanoparticles are accepted as applied substances with low toxicity in life and ecosystem (9, 10). Nanoparticles are used as nanocomposites in the preparation of nano-vaccines in combination or conjugation with biomolecules to induce appropriate immune responses and vaccination. In addition, nanoparticles are used as stimulators of phagocytic, macrophages, and dendritic cells, which is important in immunization (9). Solid nanocarriers are useful for delivering protein parts of vaccines, due to their easy entry into intestines, lymphoid, and mucosal tissues (11). Therefore, nanoparticles play an important role in the high performance of antigens and adjuvants and can increase the response of the immune system against vaccines (12).

This study aimed to provide a nano-gold conjugate with exotoxin A and evaluate its potential for production of an effective vaccine with long-lasting protection in a mouse model.

## Materials and Methods

***Bacterial sample***

*P. aeruginosa* PAO1 was purchased from Maragheh University of Medical Sciences, Maragheh, Iran, and confirmed by standard biochemical and phenotypic methods, including colony formation, gram staining, growth at 42 °C, oxidase test, and pigment production.

***Purification of exotoxin A***

Bacteria were cultured in a nutrient agar medium containing skim milk. The suspension of freshly cultured bacteria (0.4 ml) was added to dialyzed TSB medium (250 ml) containing 1% glycerol and 1 mM monosodium glutamate. It was placed in a shaker incubator with 120 rpm for 24 hr at 37 °C. Next, the bacteria-containing medium was centrifuged in 10000 g for 45 min at 4 °C. The bacteria sediment was collected and the supernatant containing toxin was stored in the refrigerator. Selective precipitation methods were used for partial purification of toxins. for this purpose, sodium citrate (0.3 M) was added to 0.1 of the liquid containing the toxin and dialyzed against 0/01 m buffer (pH = 8) in a dialysis bag at 4 °C for at least 24 hr and the buffer was replaced three times. The toxin-containing buffer was then centrifuged at 5000 g for 30 min and the containing toxin supernatant was removed. Ammonium sulfate (0.1%), at 60 %saturation with pH 8, was added to the supernatant and the obtained precipitate was dialyzed after centrifuging at 6000 rpm for 45 min. For further purification and concentration of the obtained toxin, the toxin-containing solution was centrifuged in 10000 g for 20 min at room temperature. To confirm the presence and purity of the toxin, polyacrylamide gel electrophoresis (SDS-PAGE) with 10% concentration was used and stained by the Coomassie Blue method, and finally was confirmed by western blotting method. 

***Preparation of toxoid***

For toxoid preparation, exotoxin A (5 mg) was solved in phosphate-buffered saline-PBS (10 ml), at pH = 7.2, and was mixed with sodium phosphate (0.01 mM), sodium chloride (0.15 mM), and formaldehyde (4%). The obtained compound was incubated at 37 °C for 4 days. Then, dialysis against phosphate buffer was performed for 48 hr. The detoxified toxin was sterilized using a 0.45 μm filter.

***Synthesis of spherical gold nanoparticles***

The aqueous HAuCl4 solution (3.9 ml with 1.25 g gold/liter) was prepared and then diluted 10 times. Then, a solution of trisodium citrate (1 ml with 1% weight) was added (on ice). The prepared solution was severely stirred with a Teflon magnet for 5 min and NaBH4 solution (3.9 ml with 0.02 Mm concentration) and diluted 5 times. Next, it was added dropwise into the prepared solution in an ice bath for 3 min. As a result, the color of the solution became pink immediately. The stirring was continued for another 5 hr until the solution was red (13).

***Conjugation of toxoid prepared with gold nanoparticles***

Gold nanoparticles (10 mg) was dispersed in KCl-HCl buffer (5 ml with 0.02 mM concentration) at pH 2. Each of the solutions (100 µl) containing exotoxin A (10 mg/ml) was added to the KCl-HCl buffer under stirring, which was continued for 2 hr. So, the solutions were conjugated by electrostatic forces.

***Immunization***

The Balb/C mice (aged 6 to 8 weeks) were divided into 4 groups (6 mice in each group) for immunization (Table 1). Each group was vaccinated in 0, 14, 28, and 42 days with one of the antigens (including native exotoxin A, nano-gold-exotoxin A conjugate, nano-gold, and PBS as controls). Initial injection consisted of Freund’s complete adjuvant and in the subsequent injections, Freund’s incomplete adjuvant was used. After sufficient mixing of antigens and adjuvant, they were injected subcutaneously into the anterior and posterior regions of the animals under relatively sterile conditions. All blood samples were taken one week after the last injection for quantitative and qualitative evaluation of immunization and total IgG titers. Blood samples were taken from the mice’s ocular sinus using hematocrit tubes and transferred to labeled microtubes. Serum samples were centrifuged and frozen at -20 °C to total IgG titer assay (13).

***ELISA assay***

The level of total IgG in the serum of different groups of mice was evaluated by the indirect ELISA method. First, according to the Checkerboard method, ELISA was performed with different dilutions of immune and non-immune antisera and different concentrations of antigen to obtain the best concentration and dilution conditions for antigens and antisera. Concentrations and dilutions in which the highest optical absorption of immune serum and the lowest optical absorption of non-immune serum (nonspecific absorption) were observed were considered the optimum conditions for the ELISA test. Finally, indirect ELISA was performed with a concentration of 5 μg/ml of each antigen and dilution of 1: 100 sera.

***Animal challenge***

*P. aeruginosa* PAO1 strain was used in animal challenge tests. First, the LD_50 _of the strain was determined intraperitoneally injection of different CFU of the bacterium into 7 groups of Balb/c mice. Next, 2 weeks after the last dose of vaccine, 7.5 10^7^ CFU (2×LD_50_) of bacteria was injected intraperitoneally to all groups and the mortality and survivals were recorded in challenged mice for one week (ethical code: 162307306). 

***Statistical analysis***

Data were analyzed by SPSS 21 software. Differences in the mean ELISA absorbance of each group with the mean of the control group were compared by Student’s t-test. *P*-values<0.05 was considered as meaningful difference. The ratio of live mice/total mice (survival percent) was used for the survival study.

## Results

***Extracted of exotoxin A***

**Figure 1:**
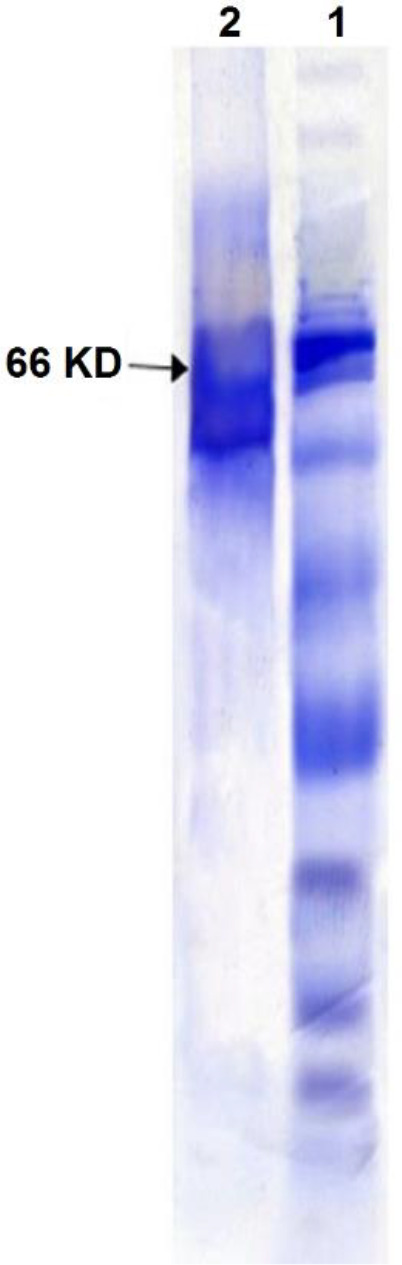
Exotoxin A varnishing electrophoresis with Coomassie Blue

The purity and presence of exotoxin A were confirmed by SDS-PAGE gel electrophoresis using the Coomassie Blue staining method. The results showed acceptable purity and quality for extracted exotoxin A (Figure 1).

***Synthesis of gold nanoparticles***

**Figure 2:**
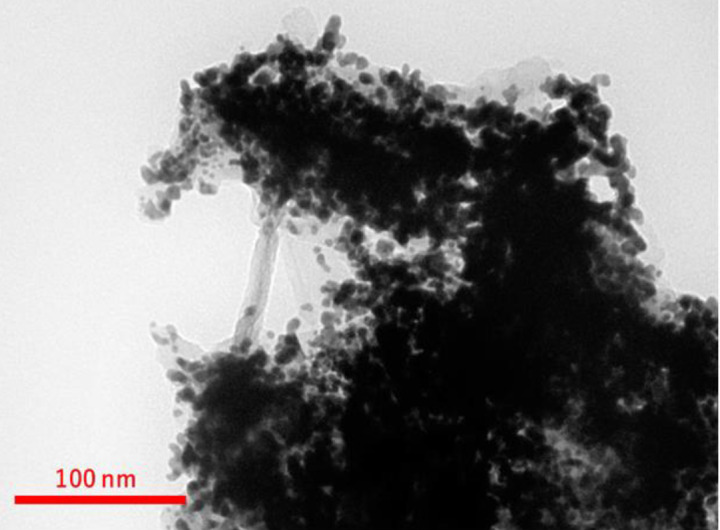
Transmission Electron Microscopy (TEM) Au-NPs gold nanoparticle-protein-free control

**Figure 3:**
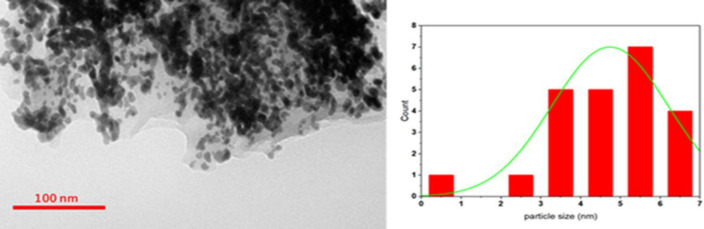
Transmission Electron Microscopy (TEM) exotoxin/Au-NPs gold nanoparticles-exotoxin control

The results of Transmission electron microscopy images (Figures 2 and 3) showed that the average size of the nanoparticles was 5 nm and the particles were almost spherical. Image mapping information precisely relevant to dispense ingredients C, N, O, P, CL, and Na confirm the correct synthesis of the desired compound.

**Figure 4:**
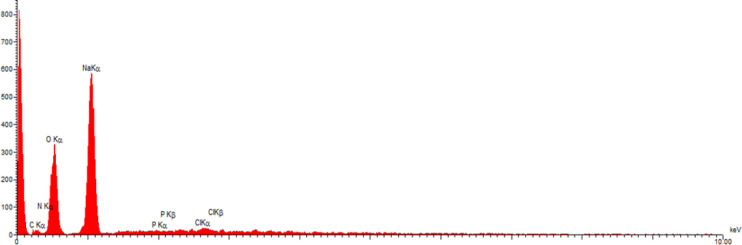
Analysis of Energy-dispersive-x-ray spectroscopy (EDX) for Exotoxin A-Nano-gold indicate. Existence of ingredients C, N, O, P, CL, Na confirms the correct structure of nano-gold- Exotoxin A

**Figure 5.**
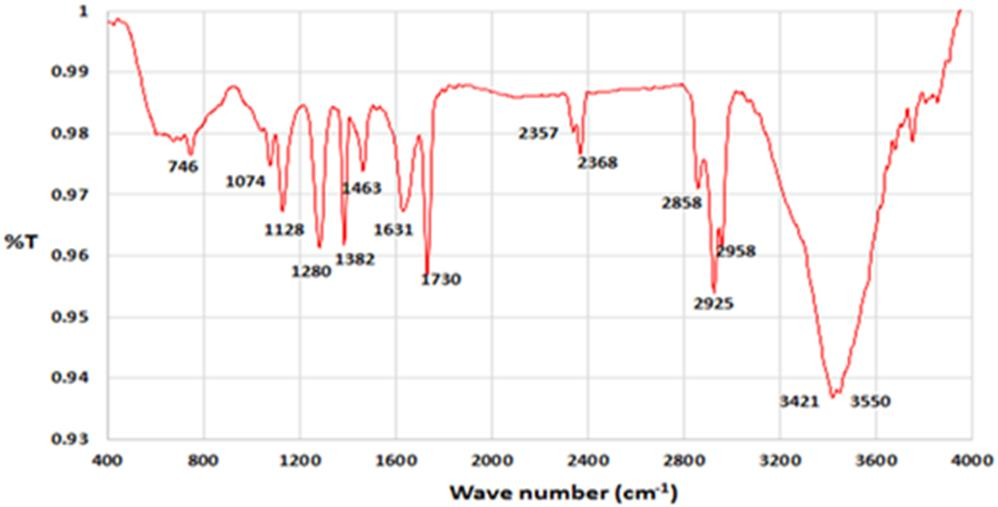
Fourier transform infrared spectrometry (FTIR) spectrum of exotoxin purification before conjugation with gold nanoparticles

**Figure 6:**
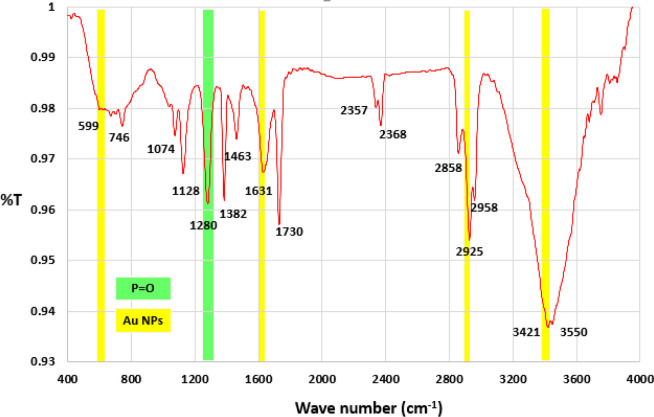
Fourier transform infrared spectrometry (FTIR) spectra of gold nanoparticles conjugated with exotoxin A

In the Fourier transform infrared spectrometry (FTIR) spectrum of native exotoxin A (Figures 4, 5, and 6), the absorption bands in the wavelengths about 1463, 1631 indicated the amino groups present in the exotoxin structure; also, the absorption bands of the wavelength 1280 indicated the p = o bond of the exotoxin structure. The absorption bands of about 1730,1382,1128 indicated the c = o groups’ presence in the exotoxin structure.

In FTIR spectrum of exotoxin conjugated with gold nanoparticles (AuNPs), a distinct absorption band appeared at about 3421, 2925, 1631, 599 showing the presence of AuNPs in the system. The results showed that AuNPs could be conjugated to exotoxin A at sizes less than 7 nm.

***ELISA***

**Figure 7:**
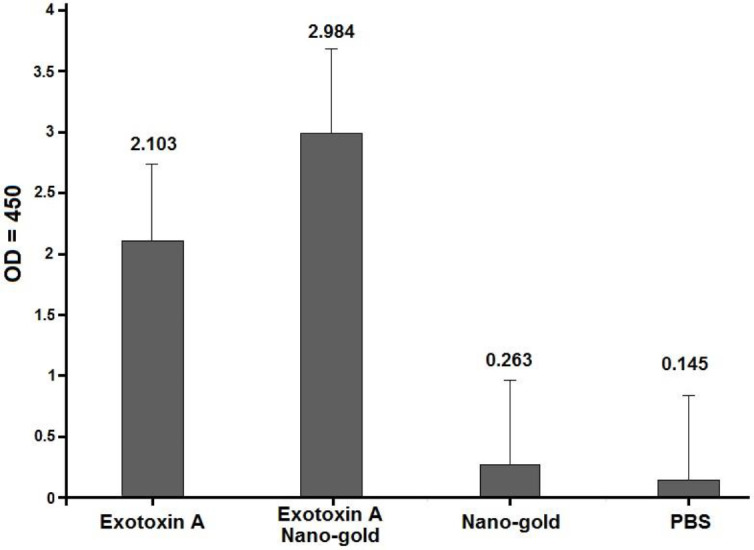
The ELISA optical density in different mice groups, one week after the last injection in 1/100 serum dilution

Absorption obtained from the serum of immune animals at a dilution of 1: 100 (at 450 nm) is shown in Table 2 and Figure 7. The amounts of antibody produced in all groups, as compared with the control, was statistically significant; the amount of antibody produced in the mice immunized with nano gold-exotoxin conjugate and AuNPs showed a significant difference with other groups.

***Animal challenges***

**Figure 8:**
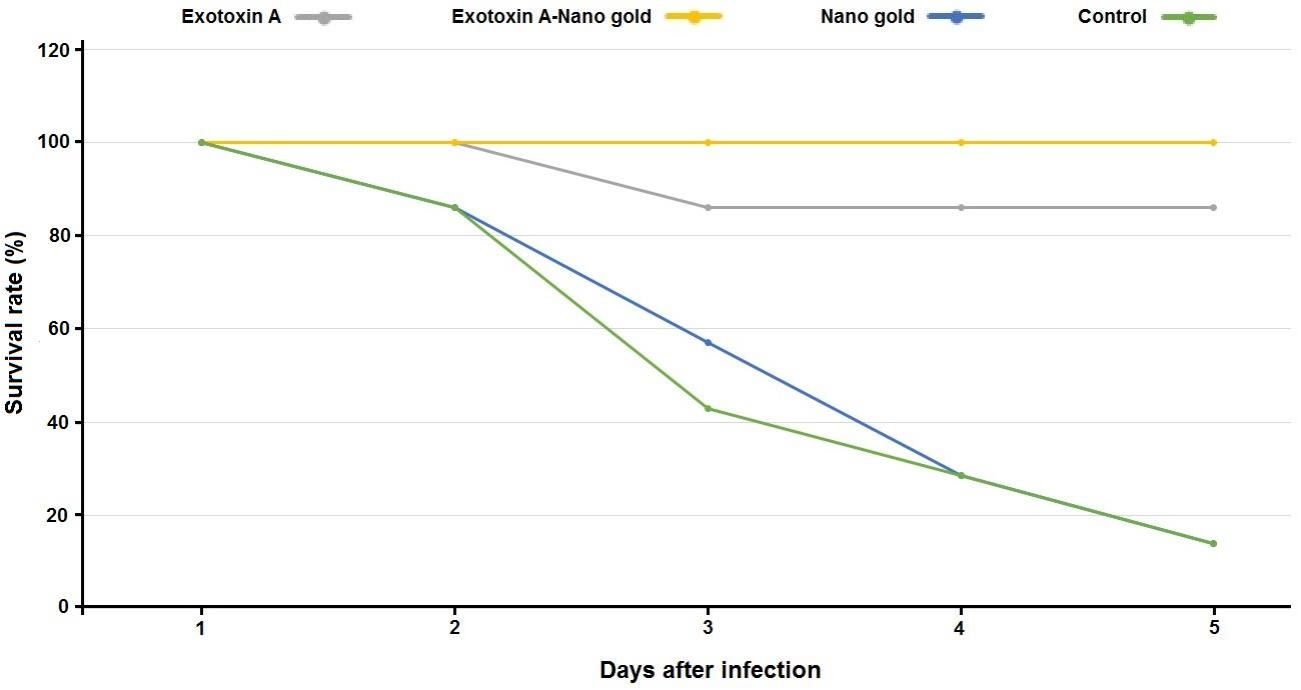
Survival rate of mice after intraperitoneal injection of *Pseudomonas aeruginosa* clinical strain with the dose of 7.5 × 107 CFU (2 × LD_50_)

The survival rate of immune animals is shown in Table 3 and Figure 8. According to these results, protection in the mice immunized by exotoxin A, conjugate, and AuNPs, was significantly different at the level of 95% confidence interval in terms of mortality and survival of the mice, as compared with the control (*P*<0.005). As could be seen in the above table, mortality rates in Exotoxin A and Exotoxin A-gold nanoparticles were significantly different from controls (nano-gold and PBS). 

**Table 1:** Antigen injection protocol in the four control and experimental animal groups

**Group**	**Antigen**	**Antigen concentration**	**Injection method**
I	EXOA + Adjuvant	37 µg	Subcutaneous
II	EXOA - Nano + Adjuvant	37 µg	Subcutaneous
II	NPG + Adjuvant	37 µg	Subcutaneous
IV	PBS + Adjuvant	300 µl	Subcutaneous

**Table 2:** ELISA optical density in different mice groups, one week after the last injection in 1/100 serum dilution

**Antigen**	**OD**_450_	**Standard deviation**	**Confidence level 95%**		**Significance level**
			**Upper limit**	**Lower limit**	
Exotoxin A	2.103	0.378	2.45	1.75	<0.005
Exotoxin A-nano-gold	2.984	0.830	3.75	2.22	<0.005
Nanogold	0.263	0.16	0.41	0.17	<0.005
PBS	0.145	-	-	-	-

**Table 3:** Survival rate of mice after intraperitoneal injection of* Pseudomonas aeruginosa* clinical strain with the dose of 7.5 × 107 CFU (2 × LD_50_)

**Antigen**	**Survival**	**Frequency**		**Variable ratio**		**Confidence level 95%**		**Significance** **level**
		**Dead**	**Alive**	**Dead**	**Alive**	**Upper limit**	**Lower limit**	
Exotoxin A	86%	1	6	14.3	85.7	1.07	0.37	0.00
Exotoxin A-nano-gold	100%	0	7	0	100	-	-	0.00
Nano-gold	14%	6	1	85.7	14.3	-	-	0.00
PBS	14%	6	1	85.7	14.3	-	-	-

## Discussion

Exotoxin A is one of the important pathogenesis factors of *P. aeruginosa* and plays a key role in bacterial immunity and pathogenicity. Cross *et al*. reported the toxin as an immunogenic factor and the difference in serum antibody titers of the patients killed by *P. aeruginosa *and the survivors of this infection. In this study, we used *P. aeruginosa* PAO1 to purify exotoxin A. This bacterium produces the highest toxin in the dialysate of trypticase soy broth (TSB) containing glycerol and monosodium glutamate. The presence of the toxin in the primary culture samples was demonstrated using SDS PAGE (agar gel electrophoresis), as compared with the standard toxin. The toxin produced was comparable in terms of molecular weight to the standard strain. By using two staining methods of silver nitrate, we showed that the toxin from the *P. aeruginosa* PAO1 strain was similar to the standard toxin.

Purification of exotoxin A from the culture medium is difficult due to the bacterial secretion of the proteases. Based on the results of our studies, purified exotoxin A can be considered as a vaccine candidate for *P.* *aeruginosa *infections. Therefore, in other studies, exotoxin A has also been considered a vaccine candidate alone or in conjugation with other compounds. Purification of exotoxin A has many problems, including its low efficiency, so in some studies, recombinant exotoxin has been considered (14-16). In this study, AuNPs were synthesized by the Turkevich method, using gold salt and sodium citrate. The AuNPs conjugated with toxin were stable and stored in the refrigerator for a long time. Weaker gravitational forces such as van der Waals, electrostatic and hydrophobic forces, bind these reactions. The AuNPs prepared in this project were less than 7 nm. AuNPs are typically about 1–100 nm in size, and most AuNPs synthesis methods are based on the reduction of gold salt composition (HAUCL4) by reducing agents.

AuNPs have been proposed as suitable candidates for drug delivery or drug carriers because of their properties, such as inactivity and non-toxicity to cells and the environment. In addition, particle dispersion (1–150 nm) contributes to the drug delivery properties of the nanoparticles. AuNPs with positive charges can bind to DNA effectively and protect enzymatic digestion (17).

In order to use AuNPs biologically, their surface must be functionalized. AuNPs are functionalized with the aim of enhancing smartness, ensuring the insensitivity of the immune system, and reducing toxicity in the body. Depending on the application of the nanoparticles, AuNPs can be functionalized with polyethylene glycol to reduce toxicity, escape from the immune system, and maintain longer blood circulation (18).

The Elisa results showed that high antibody titers against exotoxin A were produced in all mouse groups, but antibody titers produced in exotoxin A-nano-gold conjugate groups were higher than in other groups. This fact indicates that conjugation of exotoxin A with nano-gold potentiates its immunogenicity *in vivo*. Therefore, exotoxin A gold nano-particles can be considered as a potent and appropriate vaccine candidate and could act against the toxic activity of exotoxin A and pseudomonas infections. However, the use of AuNPs alone and as a conjugate with exotoxin A could be used as an efficient factor in active and passive vaccination and immunizations against pseudomonas infections. The immunogenic properties of exotoxin A, lipopolysaccharide-exotoxin A, and exotoxin A-pilin conjugates and other antigenic factors have been studied by researchers (2, 19, 20). 

Results of animal challenges showed that high protection was observed in exotoxin A, as well as with exotoxin A AuNPs conjugate groups against 2LD_50_ intraperitoneal injection of live *P. aeruginosa*. These results were similar to the results of the studies by Michalska and Wolf (21), Li *et al*. (22), and Nazari *et al.* (23). In the so-called studies, a similar protective role was reported in mice immunized with ExoA-OprF-OprI fusion groups; however, they reported poor protection in mice immunized with recombinant exotoxin A. Mortality of animals in unprotected mice including exotoxin A immunized groups showed a few days of delay in death in comparison with controls.

Various studies have demonstrated the role of anti-exotoxin A antibody in counteracting the effects of exotoxin A under *in vitro* and *in vivo* conditions (24). Therefore, the toxic effects of exotoxin A in *P. aeruginosa*, infections should be neutralized in vaccination studies. For this reason, in different studies exotoxin A with different immunogenic factors or with different nanoparticles (2, 25-27) have been investigated, alone or in conjugation, reporting different results. In this study, we used exotoxin A and AuNPs for the first time. That is why there is not much information in the literature review. Different antigens of *P. aeruginosa* have been evaluated for immunogenicity and induction of protective responses in mouse models [28]. But none of the antigens alone could induce proper protective responses. Therefore, it has been suggested that a combination of different antigens should be used to better induce effective immune responses against infections of this bacterium. Examples of these compound antigens to enhance their immune efficacy in mice include lipopolysaccharide-exotoxin A, alginate-exotoxin A, and exotoxin A with Elastase, FliC, and alkaline protease conjugates, as reported by various researchers (29). On the other hand, native purified exotoxin A is toxic to the cells, and toxoid forms may change its immunological properties, which make it not suitable for immunization purposes. On the other hand, native exotoxin A is very immunogenic, and in the serum of all patients vaccinated with exotoxin A, high titers of anti-exotoxin A antibody were demonstrated (2).

Serum antibody analysis of mice immunized with conjugated exotoxin A toxoid showed little difference with vaccination by pure exotoxin (30). These findings, confirm immunogenic features of the exotoxin A toxoid. *In vitro* and *in vivo* studies have also shown that antibody induction by non-toxic exotoxin could also be performed without cytotoxicity. Therefore, non-toxic exotoxin A can be used to create active and passive immunity to counteract the pathological effects of exotoxin A.

Finally, given the increasing worldwide antibiotic resistance especially by Gram-negatives, followed by deaths and the additional costs associated with it, the need to study the development of vaccines and other types of nosocomial infection prevention have increased. Particular attention should be paid to the costly bacteria in medical centers (31-33).

## Conclusion

We showed that AuNPs conjugated with exotoxin A have an adjuvant effect that can be used to combat a range of nosocomial infections causing by *P. aeruginosa.* In addition, the use of the toxoid has no cytotoxic effects and is also effective in providing immunity against pseudomonas-related diseases. Indeed, given the adjuvant effects and immune response potentiation, the use of AuNPs conjugated with bacterial proteins could be considered as a potential strategy for the production of microbial vaccines.

## Authors’ Contributions

This study was conceived and led by MA, AT, FKZ, and SZJ did the field experiments and collected the samples. MA, FZ, and HH analyzed the data and wrote the manuscript. All authors discussed the results and their implications and commented on the manuscript as it progressed. All authors read and approved the final manuscript.

## Funding

No external funding was required for this study.

## Conflicts of Interest

The authors declare that no conflicts of interest exist.

## References

[1] Amini B, Kamali M, Salouti M, Yaghmaei P (2017). Fluorescence bio-barcode DNA assay based on gold and magnetic nanoparticles for detection of Exotoxin A gene sequence. Biosens Bioelectron.

[2] Mahdavi S, Tanhaeivash E, Isazadeh A (2018). Investigating the presence and expression of stx1 gene in Escherichia coli isolated from women with urinary tract infection using real-time PCR in tabriz, Iran. Int J Enteric Pathog.

[3] Streeter K, Katouli M (2016). Pseudomonasaeruginosa: A review of their pathogenesis and prevalence in clinical settings and the environment. Infect Epidemiol Med.

[4] Michalska M, Wolf P (2015). Pseudomonas Exotoxin A: optimized by evolution for effective killing. Front Microbiol.

[5] Sharma A, Krause A, Worgall S (2011). Recent developments for Pseudomonas vaccines. Hum Vaccin.

[6] Hassan R, El-Naggar W, Abd El-Aziz AM, Shaaban M, Kenawy HI, Ali YM (2018). Immunization with outer membrane proteins (OprF and OprI) and flagellin B protects mice from pulmonary infection with mucoid and nonmucoid Pseudomonas aeruginosa. J Microbiol Immunol Infect.

[7] Vahedian V, Asadi A, Esmaeili P, Zamani S, Zamani R, Hajazimian S (2020). Anti-inflammatory activity of emu oil-based nanofibrous scaffold through downregulation of IL-1, IL-6, and TNF-α pro-inflammatory cytokines. Horm Mol Biol Clin Investig.

[8] Adeli M, Hosainzadegan H, Pakzad I, Zabihi F, Alizadeh M, Karimi F (2013). Preparation of silver nanoparticle containing starch foods and evaluation of antimicrobial activity. Jundishapur J Microbiol.

[9] Jadid MF, Shademan B, Chavoshi R, Seyyedsani N, Aghaei E, Taheri E (2021). Enhanced anticancer potency of hydroxytyrosol and curcumin by PLGA-PAA nano-encapsulation on PANC-1 pancreatic cancer cell line. Environl Toxicol.

[10] Luo YH, Chang LW, Lin P (2015). Metal-based nanoparticles and the immune system: activation, inflammation, and potential applications. BioMed Res Int.

[11] Raghuwanshi D, Mishra V, Suresh MR, Kaur K (2012). A simple approach for enhanced immuneresponse using engineered dendritic cell targeted nanoparticles. Vaccine.

[12] Rosenkrands I, Vingsbo-Lundberg C, Bundgaard TJ, Lindenstrøm T, Enouf V, van der Werf S (2011). Enhanced humoral and cell-mediated immune responses after immunization with trivalent influenza vaccine adjuvanted with cationic liposomes. Vaccine.

[13] Najafzadeh F, Tanomand A, Haddadi A, Majidi J (2021). Preparation and immunological properties of a nanovaccine against Pseudomonas aeruginosa based on gold nanoparticles and detoxified lipopolysaccharide. Iran J Basic Med Sci.

[14] Bayat A, Kamali A, Zarei A, Mortazavi Y, Habibi A, Amini B, Isolation, identification (2010). cloning of transfer domain of exotoxin A from Pseudomonas aeruginosa. Kowsar Med J.

[15] Tanomand A, Farajnia S, Najar Peerayeh S, Majidi J (2013). Cloning, expression and characterization of recombinant exotoxin a-flagellin fusion protein as a new vaccine candidate against Pseudomonas aeruginosa Infections. Iran Biomed J.

[16] Rana S, Bajaj A, Mout R, Rotello VM (2012). Monolayer coated gold nanoparticles for delivery applications. Adv Drug Deliv Rev..

[17] Farajnia S, Peerayeh SN, Tanomand A, Majidi J, Goudarzi G, Naghili B (2015). Protective efficacy of recombinant exotoxin A-flagellin fusion protein against Pseudomonas aeruginosa infection. Can J Microbiol.

[18] Sapsford KE, Algar WR, Berti L, Gemmill KB, Casey BJ, Oh E (2013). Functionalizing nanoparticles with biological molecules: developing chemistries that facilitate nanotechnology. Chem Rev.

[19] Lang AB, Horn MP, Imboden MA, Zuercher AW (2004). Prophylaxis and therapy of Pseudomonas aeruginosa infection in cystic fibrosis and immunocompromised patients. Vaccine.

[20] Hertle R, Mrsny R, Fitzgerald DJ (2001). Dual-function vaccine for Pseudomonas aeruginosa: characterization of chimeric exotoxin A-pilin protein. Infection and immunity.

[21] Michalska M, Wolf P (2015). Pseudomonas Exotoxin A: optimized by evolution for effective killing. Front Microbiol..

[22] Li C, Li D, Wan G, Xu J, Hou W (2011). Facile synthesis of concentrated gold nanoparticles with low size-distribution in water: temperature and pH controls. Nanoscale Res Lett.

[23] Nazari ZE, Banoee M, Sepahi AA, Rafii F, Shahverdi AR (2012). The combination effects of trivalent gold ions and gold nanoparticles with different antibiotics against resistant Pseudomonas aeruginosa. Gold Bull..

[24] Green VL, Verma A, Owens RJ, Phillips SE, Carr SB (2011). Structure of New Delhi metallo-β-lactamase 1 (NDM-1). Acta Crystallogr Sect F Struct Biol Cryst Commun.

[25] Taranejoo S, Janmaleki M, Rafienia M, Kamali M, Mansouri M (2011). Chitosan microparticles loaded with exotoxin A subunit antigen for intranasal vaccination against Pseudomonas aeruginosa: An in vitro study. Carbohydr Polym.

[26] Rai M, Yadav A, Gade A (2009). Silver nanoparticles as a new generation of antimicrobials. Biotechnol Adv..

[27] Kumar A, Jakhmola A (2007). RNA-mediated fluorescent Q-PbS nanoparticles. Langmuir..

[28] Kamei A, Coutinho-Sledge YS, Goldberg JB, PriebeGP, Pier GB (2011). Mucosal vaccination with a multivalent,live-attenuated vaccine induces multifactorial immunity against Pseudomonas aeruginosa acute lung infection. Infect Immune.

[29] Morlon-Guyot J, Méré J, Bonhoure A, Beaumelle B (2009). Processing of Pseudomonas aeruginosa exotoxin A is dispensable for cell intoxication. Infect Immune.

[30] Elzaim HS, Chopra AK, Peterson JW, Goodheart R, Heggers JP (1998). Generation of neutralizing antipeptide antibodies to the enzymatic domain of Pseudomonas aeruginosa exotoxin A. Infect Immune.

[31] Mahdavi S, Isazadeh AR (2019). Investigation of contamination rate and determination of pattern of antibiotic resistance in coagulase positive staphylococcus aureus isolated from domestic cheeses in Maragheh, Iran. Pathobiol Res.

[32] Samadi N, Pakzad I, Sefidan AM, Hosainzadegan H, Tanomand A (2015). Study of aminoglycoside resistance genes in enterococcus and salmonella strains isolated from ilam and milad hospitals, Iran. Jundishapur J Microbiol.

[33] Yari Z, Mahdavi S, Khayati S, Ghorbani R, Isazadeh A (2019). Evaluation of antibiotic resistance patterns in Staphylococcus aureus isolates collected from urinary tract infections in women referred to Shahid Beheshti educational and therapeutic center in Maragheh city, year 2016. Med J Tabriz Uni Med Sci.

